# Traditional Use of Wild Edible Plants in Slovenia: A Field Study and an Ethnobotanical Literature Review

**DOI:** 10.3390/plants13050621

**Published:** 2024-02-24

**Authors:** Andreja Papež Kristanc, Samo Kreft, Simona Strgulc Krajšek, Luka Kristanc

**Affiliations:** 1 Wild Garden Institute, Zoisova 17, 4000 Kranj, Slovenia; andrejapapez@gmail.com; 2 Faculty of Pharmacy, University of Ljubljana, Tržaška cesta 32, 1000 Ljubljana, Slovenia; samo.kreft@ffa.uni-lj.si; 3 Biotechnical Faculty, Department of Biology, University of Ljubljana, Večna pot 111, 1000 Ljubljana, Slovenia; simona.strgulc@bf.uni-lj.si; 4 Faculty of Health Sciences, University of Novo mesto, Na Loko 2, 8000 Novo mesto, Slovenia

**Keywords:** Slovenia, traditional use, wild edible plants, ethnobotany

## Abstract

No comprehensive research has been conducted on the traditional use of wild-grown edible plants in human nutrition for the Slovene ethnic area so far. In the literature on edible wild plants, authors often draw information about their use from foreign or international sources, such as books and databases, from which it is often unclear what people in different countries really include into their diet. Therefore, our purpose was to determine which edible wild-growing plant species have been used in Slovenia on a traditional basis. In our research, we gathered data using different methods. The data obtained from the literature review, i.e., the ethnobotanical literature and traditional cookbooks, were combined with those derived from the online sources and a field survey. This enabled us to create a database of 219 plant taxa encompassing more than 500 species from 62 families that are traditionally used in Slovenia. The most frequently represented families were Asteraceae, with 28 taxa, Rosaceae, with 22 taxa, Lamiaceae, with 18 taxa, Brassicaceae, with 17 taxa, Apiaceae, with 16 taxa, and Amaranthaceae, with 10 taxa. Plants are most often boiled, blanched, stewed or roasted, sometimes also baked in an oven or raw with additives, such as sour cream, sugar, salt or vinegar, but seldom fried. Selected traditional cookbooks and ethnological books provided good insight into the past use of wild plants, while an online and field survey enabled a comparison of their past and current state of use. The survey has shown that some very old wild plant recipes are still used within certain local communities, while younger people, influenced by new books about wild cuisine, are constantly introducing new plant species and recipes into their diet thereby establishing new traditions.

## 1. Introduction

**Figure 1:**
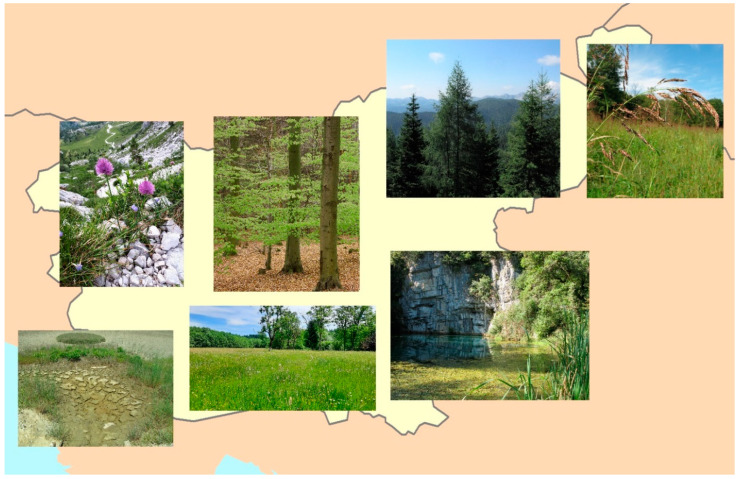
Diversity of ecosystems in Slovenia: from sea fields, steep alpine gravel slopes and spruce-larch forests to shady beech forests and various grasslands in the lowlands.

Slovenia, which is bordered by Italy to the west, Austria to the north, Hungary to the northeast, Croatia to the southeast, and the Adriatic Sea to the southwest, covers only 20,271 square kilometers but is characterized by a rich diversity of plant species, ecosystems, and landscapes (Figure 1). This diversity results from Slovenia’s transitional position at the contact area of tectonic units and biogeographical regions (the juncture of the Mediterranean Basin, the Pannonian Plain, the Eastern Alps, and the Dinaric Mountains), changing relief from sea level to high mountains, and from diverse pedological, climatic and hydrological conditions [1]. The involvement of the Slavic, German and Roman cultures in influencing human activities in the last few millennia have also contributed to the rich landscape diversity of the present Slovene territory, as well as the high variety of practices regarding plant use for consumption and herbalism [2,3].

In this respect, well-preserved forests, mountain areas and freshwater underground ecosystems with high plant diversity, including many endemic species and diverse ecosystems, are of particular importance. Fifty-eight percent of the territory is covered by forests, and approximately 25% of the territory is agricultural land in use [3,4,5]. According to estimates, approximately 60% of the environment is in a natural or seminatural state, including landscapes and areas that were managed in a traditional way in the past and where activities were abandoned a long time ago [6]. The rich cultural, landscape and plant diversity have been threatened greatly because of the pollution of surface and underground waters, soil and air, caused by the rapidly expanding industry, urbanization and agriculture in the last century. Approximately 19% of ferns and seed plants species are threatened, of which 29 are extinct, 80 are endangered, 254 are vulnerable and 257 are rare [7]. With an intention to reduce the decline in the biotic diversity many parts of the country have been classified as protected areas, such as parks, natural reserves and monuments. More than 41% of the Slovene territory is protected as national, regional and landscape parks, nature reserves or natural monuments and included in Natura 2000 sites [7]. According to the available data, there are more than 3500 registered native taxa of vascular plants and more than 850 taxa of bryophytes in Slovenia [8,9]. Their basic characteristics are derived from Alpine and Central European floristic elements and Pannonian, Dinaric and Mediterranean species. The degree of endemism (60 endemic taxa, including 22 narrow endemics with a predominant distribution in Slovenia) is considerably high in comparison to the smallness of the area [7,8,9]. Slovenian traditional medicine, which derives its knowledge in some part from the Roman and Germanic tradition, but also includes Slavic influences, uses over 500 wild-growing plants with over 6000 vernacular names [2]. Many of these medicinally important plants are also edible and were once regularly included into the diet of our people, in addition to tenths or even hundreds of others groups [2,10,11,12].

Nevertheless, the data about the traditional use of wild plant species among Slovenians is incomplete and scattered in numerous cookbooks and ethnological books. No comprehensive research has been conducted on the use of wild-grown edible plants in human nutrition for the entire Slovene ethnic area until now. In the Slovenian literature on edible wild plants, most authors often draw information about their use from foreign or international sources, such as books, ethnobotanical articles and databases, where it is most often unclear what people in different countries eat or have eaten [10,11,12,13,14]. Up until now, only a list of 218 potentially edible wild plants with no specifications of the traditionality of their use has been presented for Slovenia until now [15]. This list is not all-inclusive, as there are more than 800 potentially edible wild species in Slovenia [12]. However, our purpose was not to present the comprehensive list of potentially edible wild plant species, but to determine which of them are traditionally used in Slovenia and in what kind of dishes. Our approach was therefore similar to that chosen in the wild food ethnobotany surveys conducted in other European countries [16,17,18,19,20].

## 2. Results

### 2.1. Data Sources

**Figure 2:**
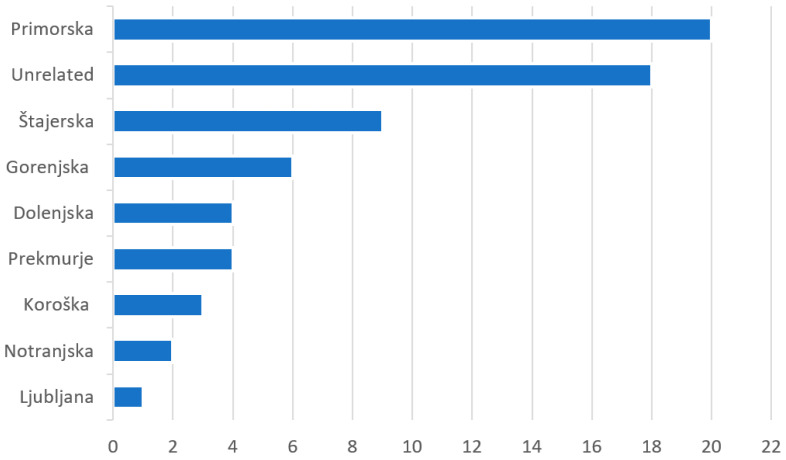
Regional affiliation of cookbooks and ethnological literature (N = 67).

**Figure 3:**
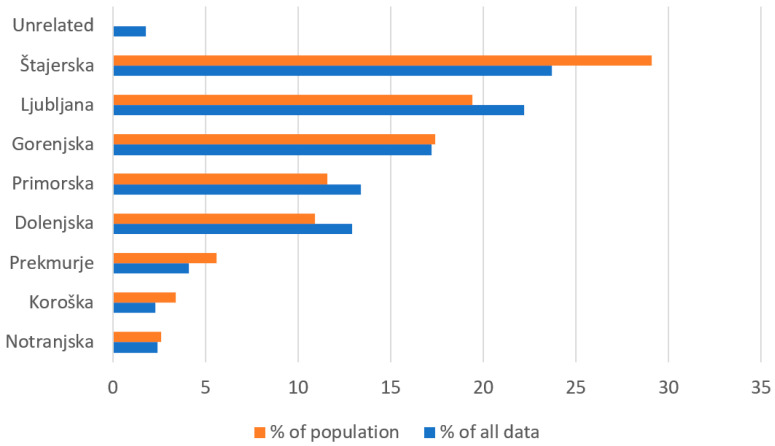
The regional affiliations of all analyzed data, including ethnological mentions, recipes in cookbooks, answers in survey and interviews (N = 4885), were compared to the statistical distribution of inhabitants among different regions (available at https://www.stat.si/obcine (accessed on 21 February 2024)).

We reviewed 67 Slovene written sources, including 41 traditional cookbooks containing 1532 recipes of the actual use of wild-growing plants and 26 ethnobotanical books or articles containing 1209 mentions of traditional wild edible plant use [2,12,21,22,23,24,25,26,27,28,29,30,31,32,33,34,35,36,37,38,39,40,41,42,43,44,45,46,47,48,49,50,51,52,53,54,55,56,57,58,59,60,61,62,63,64,65,66,67,68,69,70,71,72,73,74,75,76,77,78,79,80,81,82,83,84,85]; their regional affiliation is shown in Figure 2 and Figure 3. It should be mentioned that some of the cookbooks contained additional ethnological descriptions of the wild plants used in the diet.

The data obtained from the literature review were supplemented primarily with those derived from the online and field survey (altogether 2144 mentions of edible wild plants use), which included 132 informants (118 responses to a structured survey questionnaire and 14 in-depth unstructured interviews) of different ages (<25 years: 32%; between 25 and 50 years: 49%; >50 years: 19%), sex (female: 69%; male: 31%) and geographical affiliation (Figure 3) (see the Materials and Methods section for a detailed methodological data).

### 2.2. Analysis of the Collected Data

Almost 12% of the survey respondents use wild-growing plants on a regular basis; approximately 46% include them in their diet occasionally (at least a few times per year), while the rest use them only seldomly (less than twice a year) or never. Almost 30% of respondents likes wild plant collecting because it is usually combined with a relaxing walk in the nature, a quarter of them reported to gather plants primarily because of their taste, while 16.5% of them highlights the importance of their vitamin and mineral content. Only 1.2% reported that they use wild plants out of necessity in case of hunger.

**Figure 4:**
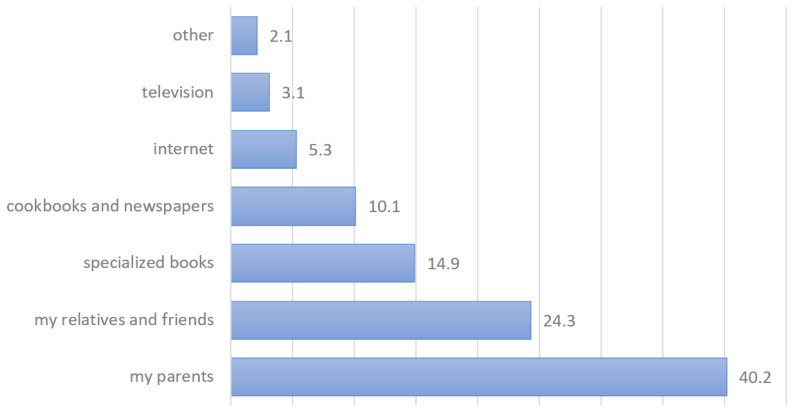
Most important sources of information about the use of wild edible plants (in %).

Most of the respondents (64.5%) were informed about the use of wild edible plants by their parents, relatives or friends, but specialized ethnobotanical books and cookbooks also seemed to be important sources of information on this topic (Figure 4). About 57% of them introduce new edible wild plants only after a thorough inspection of the existing literature, including the internet, a half of them also require a practical confirmation by an experienced person. However, nearly 17% of wild plant users introduce a new plant into their diet based only on one written or oral source. Almost 27% of the respondents wrote that they do not collect wild-growing plants introduced to them in adulthood.

A comprehensive literature review combined with a survey has enabled us to create a database of 219 plant taxa encompassing more than 500 species from 62 families that are traditionally used in Slovenia (Table 1).

**Figure 5:**
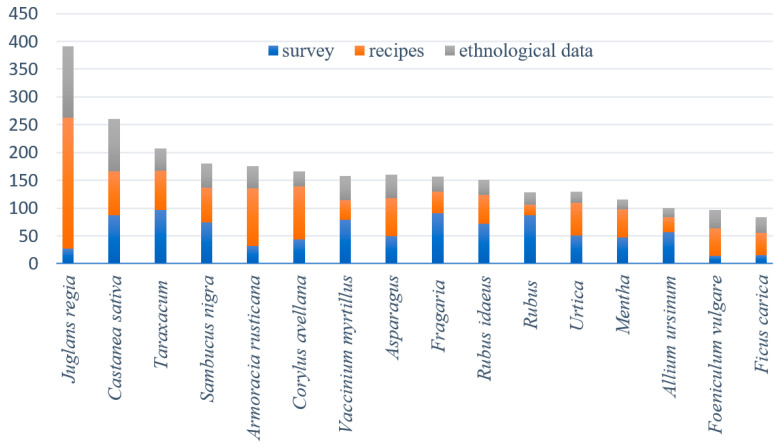
Distribution of acquired data for taxa of wild or subspontaneously growing edible plants used most frequently according to our study.

The most frequently represented family was Asteraceae, with 28 taxa, including several genera in which several species have been used traditionally. These include *Achillea*, *Taraxacum*, *Leontodon*, *Tragopogon*, *Leucanthemum* and *Artemisia*. Furthermore, Rosaceae, with 22 taxa, is also a well-represented family. Within this family, people have used several species from the genera *Rubus*, *Rosa*, *Prunus*, *Sorbus*, *Fragaria*, *Crataegus* and *Alchemilla*. Apiaceae is represented by 16 taxa and Lamiaceae by 18 taxa. The most important edible species and genera from Apiaceae are *Anthriscus*, *Carum carvi*, *Pimpinella*, *Pastinaca sativa* and *Foeniculum vulgare*, while within Lamiaceae *Mentha*, *Salvia*, *Thymus*, *Satureja* and *Lamium* are most often included in traditional dishes. There were also quite a few taxa from Brassicaceae, (17 taxa), Amaranthaceae (10 taxa), Amaryllidaceae (6 taxa), Fabaceae (6 taxa), Pinaceae (4 taxa), Polygonaceae (4 taxa), Ericaceae (4 taxa), and Boraginaceae (4 taxa). Less frequently represented families with nutritionally important species include Ericaceae with the species *Vaccinium myrtillus*, Fagaceae with the species *Castanea sativa*, Betulaceae with the species *Corylus avellana*, Juglandaceae with the species *Juglans regia* and Urticaceae with the species *Urtica dioica*. Figure 5 shows the taxa that ranked among the 10 most frequently used taxa in the survey and/or among the cookbook recipes and/or other ethnological references.

**Figure 6:**
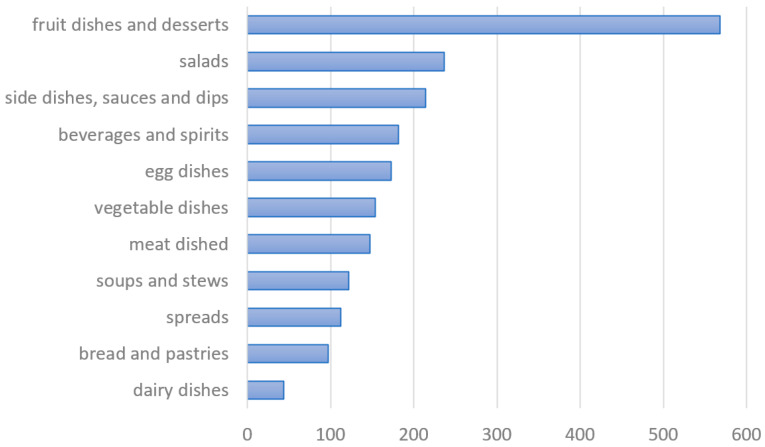
The relative frequency of types of dishes made from wild edible plants (including the data from the literature review and mentions in the survey).

As shown in Figure 6, according to the traditional cookbooks, the ethnological literature and the field survey, most recipes use either fleshy or starchy fruits or seeds and belong to the category of fruit dishes and desserts. The largest share of this category can be attributed to the use of walnuts (*Juglans regia*, 236 traditional recipes and 27 mentions the survey) and hazelnuts (*Corylus avellana*, 96 traditional recipes and 43 mentions in the survey) in desserts and, in part, to the use of chestnuts (*Castanea sativa*), figs (*Ficus carica*), raspberries (*Rubus idaeus*), wild strawberries (*Fragaria* spp.) and bilberries (*Vaccinium myrtillus*) (see also Table 1). The use of plants in other categories was more uniform; only milk (dairy) dishes and spreads seem to be rare modes of traditional wild plant use in Slovenia.

The combined analysis of the literature and the survey data has shown that asparagus (*Asparagus* spp.) has traditionally been used in soups and various vegetable and egg dishes. On the other side, horseradish (*Armoracia rusticana*) is used in meat dishes, dips, and garnishes, while the leaves of dandelions (*Taraxacum* spp.) are the most common wild ingredient of salads. In the category of meat dishes, the most important role was attributed to spices, such as rosemary (*Salvia rosmarinus*), laurel (*Laurus nobilis*), thymes (*Thymus* spp.), savories (*Satureja* spp.), juniper berries (*Juniperus communis*), and starchy chestnuts. Walnuts and mints (*Mentha* spp.) are most often used when baking bread and pastries. Laurel (*Laurus nobilis*), caraway (*Carum carvi*) and nettles (*Urtica dioica*) were found to be typical for soups and stews. On the other hand, the flowers and berries of black elders (*Sambucus nigra*), the roots of yellow gentians (*Gentiana lutea*), and bilberries, raspberries and walnuts are the most popular traditional ingredients in non-alcoholic beverages and spirits. For egg dishes, we found many recipes with elderflowers, nettles and yarrows (*Achillea* spp.), as well as with asparagus, lemon balm (*Melissa officinalis*) and mints. The survey has revealed that people often put also other wild plants in egg dishes, for instance pellitories (*Parietaria* spp.), hop shoots, elder flowers, halophytes, wild garlic (*Allium* spp.), fennel, and sometimes even chamomiles (*Matricaria* spp.). Due to the variety in the preparation of the dishes, fitting of the dish to more than one category sometimes occurred. In such cases, we developed a personal standard that we tried to follow. For example, the dish called “štruklji” could be included in “fruit dishes and desserts” category or to bread and dough dishes. Since it is more or less sweet or not sweet at all—it depends on the recipe—we decided to include all the “štruklji” dishes in the bread and dough dishes. Vegetable dishes are all meat-free dishes with vegetables as important ingredients but not eaten as salads.

**Figure 7:**
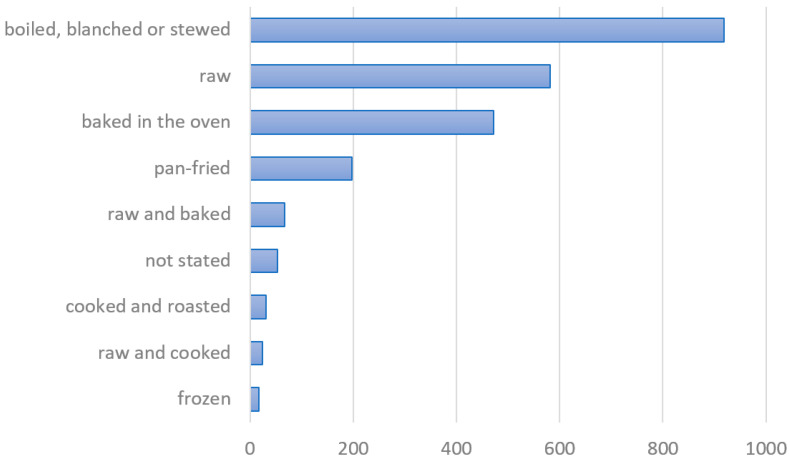
The relative frequency of the recipes containing wild edible plants according to the basic methods of preparation (including the data from the literature review and mentions in the survey).

The plants used were boiled, blanched, stewed or roasted, often also baked in an oven or raw with additives, such as sour cream, sugar, salt or vinegar, and seldom fried (Figure 7). In the past, they were usually not frozen, which is not surprising considering that freezers have been introduced only in recent decades to our country. In some recipes, two or more categories were combined (e.g., raw and baked), for example, when it was stated that a part of the plant should be baked and a part left raw to be added to the dish at the end. This is a special case in some cakes (such as bilberry or strawberry cake), where you first bake the biscuit and then add filling with raw fruits.

## 3. Discussion

### 3.1. Comparison of the Survey Data with Those Derived from the Literature

Common walnut (*Juglans regia*), horseradish (*Armoracia rusticana*), mints (*Mentha* spp.), fennel (*Foeniculum vulgare*) and the common fig (*Ficus carica*) were used significantly more often in the recipes than in the survey (Figure 5). Apart from mints, all the mentioned plants are also relatively often represented in other ethnological references, such as ethnological books and articles (see written resources in Table 1). This can be attributed to the fact that these plant species are cultivated very often, and it was often not possible to determine from the recipes or other ethnological references whether they used a wild-growing or a cultivated plant source. The survey respondents were specifically asked to enter only plants that were harvested wild; therefore, based on the low representation in the surveys, we conclude that cultivated plants of common walnut, horseradish and figs have been used mainly in Slovenia. Nevertheless, subspontaneously growing trees of common walnuts are often found in lowland forests in all regions of Slovenia, and people include their fruits in their diet. Fennel and horseradish are also either cultivated or spontaneously growing on ruderal and segetal surfaces in the majority of Slovenia. Horseradish plants grow especially abundantly in fields and in their vicinity, and their roots have also been harvested, especially during Easter. This was a common practice in the past in Slovenia [2,81], which was also confirmed by our informants, but it seems that people currently predominantly rely on cultivated sources. Similarly, people in the Primorska region have combined wild-growing and cultivated fennel in their dishes [81,82,83,84,85].

On the other hand, wild strawberries (*Fragaria* spp.), wild garlic (*Allium ursinum*), goosefoots (*Chenopodium* spp.), wormwoods (*Artemisia* spp.), chamomiles (*Matricaria* spp.), hawthorns (*Crataegus* spp.), plantains (*Plantago* spp.), common primrose (*Primula vulgaris*), hop shoots, chickweeds (*Stellaria* spp.), black locust (*Robinia pseudoacacia*) flowers, and blackberries (*Rubus* spp.) were mentioned significantly more often in the survey than in the ethnological references and recipes (Figure 5 and Table 1). We might attribute the low representation of wild strawberries in the recipes to the fact that their fruits lose their original consistency, taste and aroma very quickly and are thus most often eaten raw. Furthermore, wild strawberry picking is very time consuming, and plants are most often gathered for immediate personal use. The same applies to the use of blackberries. However, although strawberries and blackberries were not mentioned as frequently in the ethnological references as we might expect, they still ranked in the top quarter of the most frequently mentioned wild plants in this type of data source. To our surprise, wild garlic was very rarely mentioned in recipes and other ethnological references and even in newer, less traditional written sources. Therefore, we conclude that it is not used much and that it represents a new age hit in the diet of the Slovenian population [10,11,83].

Chestnut (*Castanea sativa*), dandelions (*Taraxacum* spp.), black elder (*Sambucus nigra*), blueberries (*Vaccinium myrtillus*), common hazel (*Corylus avellana*), asparagus (*Asparagus* spp.) and red raspberry (*Rubus idaeus*), as well as nettles (*Urtica* spp.), were well represented in all three types of data sources (Figure 5 and Table 1). This means that these plants have played an important role in the diet of the population in the past as well as in the present [39,43,83,84,85].

### 3.2. Traditional Use of Wild Edible Plants—A Regional Perspective

Figure 2 shows that approximately 25% of all the written sources included in this study described the recipes and eating habits of Primorska. We can see the reasons for such a marked deviation of Primorska partly in the size of the province itself but above all in the clearly more varied diet, oriented toward wild food. This is linked to the influences of the cuisines of neighboring countries (Italy and Croatia) and to the typical submediterranean climate with high plant species diversity, which includes those not growing in other parts of Slovenia. In Primorska, for example, people have traditionally used young shoots of wild asparagus, especially the species *Asparagus acutifolius*, butcher’s broom (*Ruscus aculeatus*) and common purslane (*Portulaca oleracea*) in their soups and vegetable dishes [12,43,62,64,69,84,85]; leaves of common chicory (*Cichorium intybus*), wall rockets (*Diplotaxis* spp.), pellitories (*Parietaria* spp.), glasswort (*Salicornia europaea*), sea purslane (*Halimione portulacoides*), sea beet (*Beta vulgaris* subsp. *maritima*), fennel (*Foeniculum vulgare*), mountain savory (*Satureja montana*) and rock samphire (*Crithmum maritimum*) in salads, soups, egg dishes and beverages (43,62,69,77,82,84,85]; and cornel (*Cornus mas*), strawberry tree (*Arbutus unedo*), checker tree (*Sorbus torminalis*) and true service tree (*Sorbus domestica*) fruits in jams, spirits and fruit dishes [2,12,43,44,77,84,85]) (see also Table 1).

The fact that most of the respondents were from Ljubljana and Štajerska (Figure 3) is probably the result of their large population concentration. Namely, almost 800,000 people live in Ljubljana, Celje, Maribor and the areas closely associated with them, i.e., almost 40% of Slovenians. The large share of respondents from Primorska, Gorenjska and Dolenjska (Figure 3) can be attributed partly to their dense population and large area and, possibly, to the fact that our survey reached these three regions more efficiently through the internet and interpersonal exchange than, for instance, Notranjska, Prekmurje and Koroška. As shown in Figure 3, the representativeness of these data, at least in regard to the quantity of the acquired data for different regions, is reasonably good.

Certain plant species, i.e., black elder inflorescences and fruits, horseradish roots, chestnut seeds and caraway fruits, are used in similar ways throughout the country, while some of them are used in a regionally characteristic way (Table 1). For instance, the literature often mentions the use of whitebeam (sect. *Sorbus aria*) fruits as an additive in bread baking in the past, especially during shortages in Notranjska [55,68,85], while in Štajerska (Styria) they used to bake wheat bread with added hops [52,68] and even false flax (*Camelina sativa*) seeds (see Table 1). Koroška (Carinthia) has been influenced by Austrian eating habits, such as including the fruits of lingonberry (*Vaccinium vitis-idaea*) in their jams, meat dishes and liqueurs [66]. One of the traditional Carinthian sources mentions the use of meadow sage (*Salvia pratensis*) instead of common sage (*Salvia officinalis*) [21]; however, the traditionality of the use of meadow sage in Carinthia would require obtaining more information. In Gorenjska, Notranjska and Primorska, people included hawthorn (*Crataegus* spp.) and rowan (*Sorbus aucuparia*) fruits in jams and other fruit dishes [44,55,85], which was confirmed by local informants from Dolenjska and Prekmurje, who do not stand out in terms of the frequency of use of certain taxa.

### 3.3. Transfer of Knowledge about the Use of Wild Edible Plants between Generations

Knowledge about the use of wild-growing plants is transmitted between generations predominantly through oral traditions (see Figure 4). The survey showed that more than 40% of the respondents received information about the use of wild plants through their parents, while 26% of the respondents learned about them from friends, relatives, and acquaintances (Figure 4). An interesting component, perhaps suitable for further research, is also the transfer of knowledge between children (from child to child) and between children and grandparents (from grandparent to grandchild). Grandparents have ample time, knowledge, and experience, and they often look after their grandchildren and take them for walks. In this way, many interesting plants can be introduced that were once used. Grandparents (especially grandmothers) were previously found to be an important source of traditional knowledge for their grandchildren both in Slovenia and globally [81,86,87]. Parents, of course, teach their children to use wild plants by collecting and preparing plants themselves, which many people continue even when they grow up. We believe that childhood experience is the most important, not only because of cognitive plasticity pertaining to childhood but also because of the positive attitude toward nature in general that the child develops in this way. About 25% of the respondents wrote that they do not collect wild-growing plants introduced to them in adulthood, they only considered the knowledge they had gained in childhood. An additional quarter of them considered the introduction of new plants into their diet as risky and reported that they always require practical confirmation by an experienced user or expert. We see that parents are therefore the most important transmitters of this kind of information. Specialized books on this topic (14%), cookbooks, magazines, and newspapers (12%) contributed much less to the knowledge of wild plants, while the internet and television were negligible sources in this regard (Figure 4).

### 3.4. Traditional Dishes Made from Wild Plants

Based on the data collected in the literature review and in the field survey, it has been shown that Slovenians have traditionally used wild edible plants in four ways: (*i*) as fleshy and starchy fruits and seeds, either raw or in desserts, pastries and bread; (*ii*) as leaves and young shoots in soups, stews, sauces and egg dishes; (*iii*) as spices for cooked and baked vegetables and meat dishes; and (*iv*) as an ingredient of beverages (see Table 1 and Figure 6 and Figure 7). Sometimes it was difficult to draw a line between medicinal and edible use, as in the case of linden or elderberry tea. Sometimes people drink it to improve their health, but they might do so only for thirst [12,88].

Different fruits have been included in raw fruit dishes, desserts and starchy fruits, such as bilberries (*Vaccinium myrtillus*), wild strawberries (*Fragaria* spp.) and blackberries (*Rubus* spp.), and furthermore, fruits of the true service tree (*Sorbus domestica*), blackthorn (*Prunus spinosa*), common barbery (*Berberis vulgaris*), cornel (*Cornus mas*), rosehips (*Rosa* spp.), chestnut (*Castanea sativa*) and red raspberry (*Rubus idaeus*) (Table 1). Common hazel (*Corylus avellana*), common walnut (*Juglans regia*) and common fig (*Ficus carica*) fruits were eaten just as often raw as in various baked desserts. The leaves of mints and fruits of walnuts and whitebeams were the most characteristic for bread dishes and pastries. Mints are an ingredient in the preparation of bread and sweet-salty pastries, such as “metovka” and ”štruklji” [21,68,69,70], and whitebeam and walnut fruits as an ingredient in bread and ”potica” [44,45,68,70,84,85] (Table 1).

The most common taxa in the salad category were wild garlic (*Allium ursinum*), corn salads (*Valerianella* spp.), harden rocket (*Eruca sativa*), wall rockets (*Diplotaxis* spp.), common primrose (*Primula vulgaris*) and dandelions (*Taraxacum* spp.). In egg dishes, nettles (*Urtica* spp.), lemon balm (*Melissa officinalis*), black locust (*Robinia pseudacacia*), yarrows (*Achillea* spp.), bladder campion (*Silene vulgaris*) and feverfew (*Tanacetum parthenium*) were often included [32,62,67,85]. Mostly, the egg dishes involved the preparation of the so-called ”frtalja”—an omelette with a plant material as the main ingredient, eggs and flour only serving as a binder. Young shoots of asparagus (*Asparagus* spp.) and common hops (*Humulus lupulus*) were typically used to prepare various vegetable dishes. Cranberries (*Vaccinium vitis-idaea*), the roots of the common horseradish (*Armoracia rusticana*) and fruits of the European crab apple (*Malus sylvestris*) were typical for the preparation of side dishes, sauces, vinegar and dips [12,43,85].

As expected, spices are most often used in the preparation of meat dishes in a similar way as in other Mediterranean countries [16,18,89]. These spices are common sage (*Salvia officinalis*), common juniper (*Juniperus communis*), thymes (*Thymus* spp.), true laurel (*Laurus nobilis*), rosemary (*Salvia rosmarinus*), mountain savory (*Satureja montana*) and fennel (*Foeniculum vulgare*) [43,84,85].

Bilberries, fruits and flowers (or inflorescences) of black elder (*Sambucus nigra*), cornel (*Cornus mas*) fruits, flowers of lindens (*Tilia* spp.) and leaves of plantains (*Plantago* spp.) were typically used to prepare different types of drinks, such as teas, smoothies and raw juices. The roasted roots of chicory (*Cichorium intybus*) were mainly used as a substitute for coffee, while the spruce tips and the inflorescences of dandelions were used for syrups. Wormwood (*Artemisia* spp.) and juniper (*Juniperus* spp.) berries were mostly used in the preparation of spirits.

### 3.5. Slovenian Wild Cuisine: From Traditional Use to Modern Perspectives

Before the 19th century, Slovenian people used certain types of wild plants on a wider scale, which were later replaced by other cultivated plants. This applies, for example, to chickpeas (*Lathyrus* spp., especially *Lathyrus sativus*), and different types of sorrel (*Rumex* spp.) [2,12,28,90]. Our ancestors enriched their diet, based on cereals and legumes, with weed species that thrived on cereal fields and ruderal habitats; for instance with poppy seeds and greens of wild plants of genus *Brassica*, peppercresses (*Lepidium* spp.), hedge mustards (*Sisymbrium* spp.), goosefoots (*Chenopodium* spp.) and amaranths (*Amaranthus* spp.) [12,19,28,68,90]. In the 17th and 18th centuries, some wild or cultivated plants gradually stopped being used; probably because they were replaced by tastier ones. Alexanders (*Smyrnium* spp.) were completely replaced by celery in European cuisine by the 18th century. Our field survey has shown, that although perfoliate alexanders (*Smyrnium perfoliatum*), still grows in dry karst grasslands in Slovenia, its edibility is nowadays not known even to local people. The use of bitter wild herbs, such as ground-ivy (*Glechoma hederacea*), common heather (*Calluna vulgaris*) and wormwoods (*Artemisia* spp.) in beer production had a similar fate. Slovenians have used almost exclusively hops (*Humulus lupulus*) with spice beer in the last two or three centuries, but in accordance to the findings of our survey the traditional bitter herbs, especially ground-ivy, wormwoods and even minths have gained some recognition among younger beer brewers in the last 20 years.

The interviews with older informants revealed that only a few decades ago the coffee substitutes were still sometimes prepared from the roasted roots of common chicory (*Cichorium intybus*), seeds of vetchlings (*Lathyrus* spp.) and chestnut (*Castanea sativa*), and acorns of various oak species in our country mainly from the durmast oak (*Quercus petraea*) and the pedunculate oak (*Quercus robur*). This practice is also well known from the ethnobotanical literature [2,28,69]. Very tasty and valued oil was pressed from the beech nuts. During the Second World War and in the following years, hundreds of thousands of kg of beech nuts were collected annually for this purpose in Slovenia (100 kg yields about 10–12 L of oil) [2,55,68].

Until the Second World War and for some time after it, wild plant gathering and mushroom picking was a very important additional or even the only source of seasonal income, especially among the people of lower social classes and small farmers [47,53,56,74,79]. In the last decades, in conjunction with socio-economic development, the general need for wild plant gathering gradually declined [56,74].

Due to changes in the way of life, certain plants that used to be consumed mainly by children, e.g., cornel, barbery and blackthorn fruits, are nowadays used much less often. On the other hand, some practices, such as the collection of the fruits of wild caraway (*Carum carvi*), were almost entirely lost due the degradation of the lowland grasslands. Nowadays, people gather wild plants for food and medication primarily for their personal use and not for sale [10,11,80]. From the online and field survey, we found out that the once popular fruits of cornel (*Cornus mas*), true service tree (*Sorbus domestica*), elderberry (*Sambucus nigra*), and wild caraway (*Carum carvi*), as well as the leaves of the corn salads (*Valerianella* spp.), have been partly replaced by wild garlic (*Allium ursinum*), salad burnet (*Sanguisorba minor*), yellow cresses (*Roripa* spp.), amaranths (*Amaranthus* spp.), knotweeds (*Polygonum* spp.), garlic mustard (*Alliaria petiolata*), avens (*Geum* spp.), rampions (*Phyteuma* spp.), bellflowers (*Campanula* spp.), oxtongues (*Picris* spp.), galant soldiers (*Galinsoga* spp.) and stinking aposeris (*Aposeris foetida*), as well as other plants introduced to us by the authors of books on edible wild plants [10,11,12,13,83]. The use of wild plants that grow in large numbers and have a great taste is very common among people and has also gained recognition in haute cuisine [12,13,91,92,93].

Nevertheless, the interviews with the older informants from different parts of the country confirmed that many old wild recipes are still alive among people, such as the use of roots of broad-leaved sermountain (*Laserpitium latifolium*) in spirits in Posavje and Kozjansko (Figure 2, regions 11 and 12), the use of fruits of common whitebeam (*Sorbus aria*) and hawthorns (*Crataegus* spp.) in jams in certain parts of Notranjska, the use of the checker tree (*Sorbus torminalis*) fruits in the preparation of fruit dishes in the Dragonja river valley, and the use of the common glasswort (*Salicornia europaea*) and seepweed (*Suaeda maritima*) shoots in soups and salads by inhabitants of the Sečovlje salt flats, Izola and Strunjan [12,82]. The shepherds on Velika planina alpine pastures use peculiar ingredients to make their mountain refreshing tea; for instance, thymes, lady’s mantles (*Alchemilla* spp.), common kidneyvetch (*Anthyllis vulneraria*) and even hairy alpenrose (*Rhododensron hirsutum*), named “gričevnik”, and mountain everlasting (*Antennaria dioica*), called “griževnik”. The inhabitants of Čičarija still sometimes use the fruits of the Christ’s thorn (*Paliurus spina-christi*) as an ingredient in teas and bread [84]. On the other hand, the knowledge about the use of mountain lovage (*Ligusticum mutellina*), sweet cicely (*Myrrhis odorata*) and spignel (*Meum athamanticum*) in spirits and as a spice for vegetable dishes, which were popular in the past by the inhabitants of the Julian Alps surrounding Bohinj and Kranjska Gora, is becoming obsolete. Similarly, the people forgot the practice of including raw corms of spring crocus (*Crocus vernus*), called “kroketi”, in their diet, a habit present in certain parts of Primorska in the beginning of the 20th century.

### 3.6. Protection Strategies for the Conservation of the Vulnerable Edible Plant Species

Many edible and medicinal plant species could be endangered by harvesting [14]. Picking fruits or above-ground parts of the shoots of perennials in moderate quantities is seldom problematic, unless it includes rare and endangered species. In this category, there are many species that grow near water, e.g., water nut (*Trapa natans*), on the fens, e.g., bogbean (*Menyanthes trifoliata*), bog bilberry (*Vaccinium uliginosum*) and lingonberries (*Oxycoccus* spp.), on moist meadows, e.g., pennyroyal (*Mentha pulegium*) and great burnet (*Sanguisorba officinalis*), in dry habitats, e.g., hyssop (*Hyssopus officinalis*), common sage (Salvia officinalis), snowy mespilus (*Amelanchier ovalis*, houseleeks (*Sempervivum* spp.), European nettle tree (*Celtis australis*, common smilax (*Smilax aspera*) and butcher’s-broom (*Ruscus aculeatus*), or in salt coastal marshes, e.g., rock samphires (*Chrithmum maritimum*), common glasswort (*Salicornia europaea*), saltworts (*Salsola* spp.) and sea beet (*Beta vulgaris* subsp. *maritima*). From the nature conservation point of view, the collection of the underground parts of perennial plants can be much more problematic, even unacceptable [12,43,83,85], since they cannot survive without them. In addition, the extensive collection of flowers or whole parts of annual plants could pose a threat to their populations [12,14,43].

Some plant species are endangered simply because they are present only in a narrow geographical area (so-called endemics) or because wherever they occur, they form very rare populations. Such species can be driven to extinction by even one major reckless human intervention [12,83]. As already mentioned, species that thrive in threatened habitats, such as marshes, bogs and dry grasslands, are also under great pressure. In this respect, the most important conservation measures are oriented towards habitat protection by the minimization of tourism and agriculture in these regions. Plant species in these habitats should not be harvested. The same applies to protected species, whose gathering is sometimes possible to the extent specified in the legal documents governing that area.

Therefore, when collecting, we should always ask ourselves about our purpose for collection only as much as we need and in places where there are enough plants [12]. When harvesting, we always adhere to the principle that we only take the parts of the plant that we need, we do not tear or pull them all over. This especially applies to the collection of underground parts, flowers and seeds, as in this case their regenerative capacity is lower than in the case of the collection of leaves, shoots and twigs. When picking flowers or inflorescences, we should leave at least half of them on the plant, and the same applies to the pods. It will be useful if some of the seeds and fruits are scattered around the surrounding area or even buried in the ground in suitable places [12,83]. In each plant population, only a small proportion of specimens may be taken in one season.

## 4. Materials and Methods

In this ethnobotanical research, we collected information about the use of wild edible plants from ethnological sources and selected traditional cookbooks, and conducted an online and field survey, that included informants from different parts of Slovenia.

We reviewed data from the literature, such as ethnobotanical articles, traditional cooking and ethnobotanical books and local internet sites or databases (see Table 1 and References). Only reliable traditional cookbooks were taken into consideration, while the newer cookbooks addressing new and fashionable cuisine inspired by international sources were excluded. The recipes from the traditional cookbooks with no confirmation of use among local people were not included in the analysis.

We focused only on wild plants that were used as food, i.e., for salads, sauces, garnishes, drinks and seasoning or aromatizers. This means that we did not collect data on the use of wild plants for medicinal purposes. When plants were referred to only with vernacular names, the identification of taxa was performed using glossary terms at the end of the cookbooks (when they existed), ethnobotanical books, and/or the data available in different databases, such as the database in the article of Praprotnik [94] or “Leksikon rastlinskih bogastev” [95].

The data obtained from the literature review were supplemented with those derived from the online and field survey. The survey questionnaire was initially distributed personally via an e-mail to reach some of the informants. Afterwards, in 2009, the survey was put on “Kulinarična Slovenija”, the largest culinary portal at that time. In 2023, the survey questionnaire was also sent to the professional higher education study program students at the Faculty of Health Sciences in Novo mesto and to general practitioners from all parts of Slovenia. 

In addition to some basic demographic questions, a combination of closed-type and open-type questions were included in the survey questionnaire. The questions were as follows (in case of the closed-type questions possible answers are also stated):

1. How often do you gather edible wild plants? (one choice: regularly; occasionally, i.e., several times per year; seldomly, i.e., twice a year or less; never).
2. Which wild plants do you gather on a regular basis? Include also beverages, such as teas and spirits, if they are not used for medicinal purposes.
3. Please, name any other wild plants that you remember collecting or trying at any time. You can use the list of 120 edible plants to assist your memory.
4. How did you get information about the edible wild plants you use? (multiple choices: from my parents; from friends, relatives, and acquaintances; in specialized books; in cookbooks and newspapers; on the internet; on television; other)
5. From how many sources do you inquire about the edibility of a specific plant species before introducing it into your diet? (one choice: one source is enough, either written or oral; always use multiple sources, also ethnobotanical books and internet; always require a confirmation from an experienced person).
6. For what purpose do you include the wild edible plants into your diet? (multiple choices: due to their good taste; because they are free; due to their high mineral and vitamin content; because gathering is combined to a walk in the nature; out of necessity in case of hunger; other).

At the end of the survey, 120 edible plants were listed (with a Slovenian name) to assist the respondents. Sometimes the taxa could be determined at the species level and in other occasions only at the level of genus. If the answer was ambiguous regarding either the taxonomy, the culinary use or the traditionality, the data were not included in the analysis. Sometimes, we could not identify plant taxa from vernacular names, listed by respondents, such as “hrastovka” or “dragoncelo”.

Furthermore, in-depth unstructured interviews regarding the wild edible plant use were conducted with some experienced informants (their average age was 78.4 years), i.e., herbalists, wild food experts, botanically oriented tourist guides, older sustainable farmers, wild food vendors in markets etc., from different regions of Slovenia.

**Figure 8:**
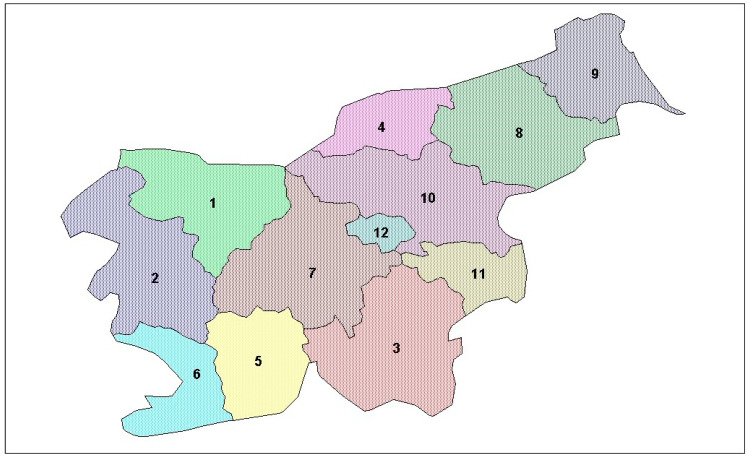
The depiction of Slovenian regions as used in this study: Dolenjska (3 and 11), Gorenjska (1), Primorska (2 and 6), Notranjska (5), Koroška (4), Štajerska (8, 10 and 12), Prekmurje (9) and Ljubljana with its suburbs (7).

Since certain parts of Slovenia can be regarded as cultural–historical entities and have specific biological and geographical characteristics, we divided Slovenia into generally known regions. These include Dolenjska, Gorenjska, Primorska, Notranjska, Koroška, Štajerska and Prekmurje (Figure 8). Due to the degradation of the environment in Ljubljana, its central location and because an increasing number of people from all over Slovenia are immigrating into the capital city, bringing their own customs with them, we have considered it and its suburbs special areas.

## 5. Conclusions

Wild plant gatherings for food and fodder have always been a part of people’s lives in our country. In the newspapers and books from the 19th and 20th centuries, we can find many interesting facts about the selection of wild plants used among Slovenians [26,28,63,68,69,70,71,83,90], while the literature for previous centuries is less well preserved. Different sources of information (traditional cookbooks, ethnological books, surveys, and interviews) provide different information about the types of wild plants and their use in food. Selected traditional cookbooks, ethnological books and interviews provided good insight into the past use of wild plants and an online and field survey into their past and current state of their use. During the course of this study many older informants were interviewed giving information about the use of wild plants in the decades after the Second World War. They were also a good source of information about the reliability of the traditional use of the recipes in the cookbooks.

Slovenians are still connected to a considerable extent with forests and meadows and some of their products, which are sometimes provided in large quantities. Our connection with wild plant food has also been proven by the survey, as almost 46% of respondents collect wild plants often or at least several times a year, which is comparable to the results of similar wild food ethnobotany surveys conducted in Europe [16,18,19,91]. There are slightly fewer (approximately 29%) who collect these plants only once or twice a year or occasionally (14%). Nevertheless, those who consider wild plants to be an important part of their daily diet and therefore collect them very often (12% of respondents) are also rare. Interestingly, the popularity of some wild plants in the diet of our people has changed over time. Tens of new wild plant species have been introduced widely by the authors of books on edible wild plants [10,11,12,13]. Our survey has shown that the use of plants that grow in large numbers, that are easily accessible and also tasty, e.g., bilberries, wild strawberries, chestnuts, dandelions and wild garlics, is very common among Slovenian people. This is probably positive from the health perspective and is sustainable, since their populations are abundant and could thus not be easily endangered. However, many other plants are more or less ignored, remaining in the domain of those who are more in touch with nature, enthusiasts and experimenters (see species mentioned only once or twice in Table 1).

## Figures and Tables

**Table 1:** List of edible wild plants used traditionally in Slovenia.

Botanical Family (APG IV System)	Scientific Name	Slovenian Vernacular Names **	Data Quantity C/E/S *	Parts Used/Typical Dishes	Regional Affiliation	Written Sources—References
Adoxaceae	*Sambucus nigra* L.	bezgovina, bezgovc, beza, bzg, bažovina, boz, zobovec, zovina	63/43/74	fruits and flowers/beverages, spirits, egg and fruit dishes	/	[21,22,26,27,29,30,31,39,41,42,45,46,50,51,52,55,60,62,63,66,67,69,70,71,72,74,77,78,84,85]
	*Sambucus racemosa* L.	divja beza, češuljek, rdeči bezeg	0/0/1	fruits/spirits	/	/
	*Viburnum opulus* L.	kozja pogačica, kalina, kačji les, kalinovec, tršljika, žgalinovec	0/2/0	fruits/beverages, jams	/	[2,43]
Amaranthaceae (incl. Chenopodioideae)	*Amaranthus* L.	ščer, amarant	0/1/0	seeds, young shoots/bread, soups, salads	/	[28]
	*Atriplex* L.	loboda	1/5/0	leaves/sauces, garnishes	Primorska	[65,82,83,85]
	*Beta vulgaris* (L.) Arcang.	primorska pesa	0/3/0	young shoots/salads, soups, egg and vegetable dishes	Primorska	[12,43,85]
	*Chenopodium* L. (*C. album* L. and *C. hybridum* L.)	škrobla, bela metla, loboda, beli kozji rep	3/2/12	leaves/sauces, garnishes	Primorska	[43,65,85]
	*C. polyspermum* L.	mnogosemenski kozji rep	0/0/1	leaves/soups	/	/
	*Chenopodium bonus-henricus* L.	dobri jurko, kopjasti kozji rep, kozji rep	0/0/1	leaves/soups, spreads	Gorenjska	/
	*Halimione portulacoides* (L.) Aellen	tolščakasti lobodovec, tolščakasta loboda, morska loboda, omahlina	0/3/0	young shoots and leaves/salads, soups, egg and vegetable dishes	Primorska	[12,43,85]
	*Salicornia europaea* L.	osočnik, zelnati osončnik, salikornija, omaga, omakalj	1/4/1	young shoots/salads, soups, egg and vegetable dishes	Primorska	[12,43,82,85]
	*Salsola* L. (especially *S. soda* L)	solinka, slanica	0/2/0	young shoots/salads, soups, egg and vegetable dishes	Primorska	[12,43]
	*Suaeda maritima* (L.) Dum.	obrežna lobodka, primorski slanorad	0/2/1	young shoots/soups, vegetable dishes	Primorska	[43,85]
Amaryllidaceae	*Allium* L.	luk	5/3/10	aerial parts, bulbs/soups, salads, spreads, garnishes	/	[36,41,55]
	*Allium ampeloprasum* L.	poletni luk	3/4/2	aerial parts/soups, spreads	/	[36,42,55,78]
	*Allium angulosum* L.	robati luk	0/0/1	aerial parts/salads, soups	/	/
	*Allium schoenoprasum* L.	drobnjak, sibirski luk, drobnik, šintelc, šnitlah	0/1/2	aerial parts/soups, spreads, garnishes	Gorenjska	[36]
	*Allium ursinum* L.	čremož, divji česnik, gozdni česen, kačji lek, štrkavec	27/16/56	aerial parts/soups, salads, spreads, egg dishes	Ljubljana	[32,36,41,42,55,78,85]
	*Allium victorialis* L.	vanež	0/0/1	aerial parts/salads	/	/
Apiaceae	*Aegopodium podagraria* L.	regačica, samojeja	0/2/4	young leaves/soups, vegetable dishes	Gorenjska	[42,43]
	*Angelica sylvestris* L.	gozdna angelika, kadunec, ilka, kravojec	0/2/1	roots, fruits/beverages	/	[2,43]
	*Anthriscus* Pers.	krebuljica, francoski peteršilj	1/1/2	leaves/soups, vegetable dishes	/	[42,43]
	*Carum carvi* L.	čumna, bela cemena, kimca, kimelj, kim, divji kumen, kumič, kumin	52/17/10	fruits and leaves/sauces, garnishes, spirits, soups, vegetable, milk, meat and egg dishes	/	[21,24,27,29,31,36,38,41,42,48,49,50,57,60,66,69,85]
	*Crithmum maritimum* L.	morski koprc, motar, matar. Ščulec, petrovo zelje	2/2/5	leaves/salads, pickled, sauces	Primorska	[43,60,69,85]
	*Daucus carota* L.	koren, kuzmorka, mrkelca, merlin, svinjski koren, merlen, korenje	1/3/4	roots/soups and sauces	Ljubljana	[42,55,85]
	*Ferulago campestris* (Besser) Grecescu	koromačnica	0/1/1	young leaves/soups, vegetable dishes	Primorska	[43]
	*Foeniculum vulgare* Mill.	komarček, fenkelj, morač, fidoči, koprc, koromač, morski janež	49/33/14	fruits, inflorescences and leaves/sauces, garnishes, spirits, soups, vegetable, fruit and egg dishes	Primorska	[21,22,31,32,36,38,41,44,45,48,57,60,62,64,65,67,69,84,85]
	*Heracleum sphondylium* L.	bršč, medvedove tace, konjska kumna, moška moč	0/3/3	young leaves/soups, vegetable dishes	/	[12,42,43]
	*Laserpitium latifolium* L.	jelenovec, košutnik	0/2/2	roots and fruits/beverages	Štajerska	[12,43]
	*Ligusticum mutellina* (L.) Crantz.	velestika, veleždin, ljubačac, lesandrina, milobud, miloduv, selim	0/1/1	young leaves/soups, vegetable dishes	Gorenjska	[43]
	*Meum athamanticum* Jacq.	planinski štrbec	0/0/1	leaves, fruits/desserts, beverages	Gorenjska	/
	*Myrrhis odorata* (L.) Scop.	kromač, dišeča krebulca	2/2/3	leaves and fruits/syrups, beverages, desserts	Gorenjska	[42,43,55]
	*Pastinaca sativa* L.	rebrinec, pastinak, pastinaka	4/3/6	roots/soups, vegetable dishes	/	[35,36,43,45,85]
	*Pimpinella* L.	bedrenec, bibernelica	0/3/2	leaves and fruits/syrups, beverages, desserts	/	[12,43]
	*Smyrnium perfoliatum* L.	repušica	0/2/0	young leaves/soups, vegetable dishes	Primorska	[12,43]
Araceae	*Arum italicum* Mill.	štrkat, zminac, zmijinac	0/2/0	rhizoma/spirits, cooked	Primorska	[43,85]
	*Arum maculatum* L.	kačjak, kačje zelje, kačnik, kozlac	0/2/0	rhizoma/cooked	Primorska	[43,85]
Asparagaceae	*Asparagus* L.	beluš, beluša, pojanica, pojavec, špargelj, smrečica	69/50/40	aerial parts/soups, spreads, egg, vegetable and meat dishes	Primorska	[21,31,32,36,41,42,45,52,60,63,64,65,69,71,85]
	*Ruscus aculeatus* L.	lobodika, bodeča mlejzek	2/4/1	young shoots/egg dishes, vegetable dishes	Primorska	[50,60,65,85]
Asteraceae (incl. Cichorioideae)	*Achillea* L.	jezičec, arman, hrman, kačjek, raja, repiček, stolistnik, škarucelj, škrebec, romancvet, škoreča	8/6/29	aerial parts/soups, egg dishes, beverages, spirits	Primorska	[27,29,32,44,45,50,51,60,63,70,85]
	*Achillea clavenae* L.	korocvet, skorecelj	0/3/0	aerial parts/fermented beverages	Primorska	[60,68,69]
	*Antennaria dioica* (L.) Gaertn.	griževnik, majnica	0/0/1	inflorescences/teas	/	/
	*Aposeris foetida* (L.) Cass. ex Less.	gozdni regrat, krompirjevka	0/4/7	leaves/salads	Ljubljana	[12,42,55]
	*Arctium* L.	kloš, kencelj, reginec, repivec, repje, starec, torica, torika, podbev, lepenj, lepir	0/3/0	roots/soups, vegetable dishes	/	[12,43,85]
	*Arnica montana* L.	brdnja, aronk, črnivec, gorski kokovičnik, gorska svetlica, moravka, zlatenica, maerni koren volčji zob	0/9/6	aerial parts/beverages	/	[2,82,83]
	*Artemisia* L.	pelin	2/2/2	aerial parts/spirits	/	[2,42,67]
	*Artemisia absinthium* L.	grenki pelin	3/4/13	aerial parts/spirits	Primorska	[42,56,69,70,85]
	*Artemisia vulgaris* L.	komoljika	2/3/8	aerial parts/spirits	Primorska	[42,56,67,70]
	*Bellis perennis* L.	marjetica, iskrica, rigelc, ranik, mezinčica, kostanjička, katarinčica, micike, mičkice, margaretica	2/4/5	leaves/salads, beverages, garnishes	Gorenjska	[42,55,82,85]
	*Carlina acaulis* L.	bodeča neža, bržavda, bodič, kampaneža, neževje, sitnec, skočnjak, veliki striček	0/1/1	young flowerhead bud/salads, vegetable dishes	/	[85]
	*Chondrilla* L.	hrustavka	0/1/1	leaves/salads	/	[85]
	*Cichorium intybus* L.	potrošnik, cikorija, jedrik, mleč, petrovčnik, ozlika, podvornica, popotnik, radič	20/16/7	leaves and roots/salads, spreads, garnishes, soups, egg dishes, beverages	Primorska	[22,27,48,49,59,60,61,63,64,65,69,85]
	*Cirsium oleraceum* (L.) Scop.	sršje, vodenika	0/3/1	roots, young leaves/salads, soups, spreads	/	[2,43,83]
	*Helianthus tuberosus* L.	papežica, sladki krompir, topinambur, laška repa, papeževa repica, gomoljasta sončnica	0/4/4	tubers/vegetable dishes	/	[12,43,82,85]
	*Helichrysum italicum* (Roth) Loudon	smilje, madronščica, božja strešica, magriž, malagriž	0/2/0	leaves, inflorescences/soups, vegetable, meat and fish dishes	Primorska	[43,85]
	*Hypochaeris* L. (especially *H. radicata* L.)	svinjak, trpežni svinjak	0/2/1	leaves/salads, soups, spreads	/	[12,43]
	*Lapsana communis* L.	kolenček, kolenče	0/0/1	leaves/salads	/	/
	*Leontodon* L.	jajčar, otavčič, žoltenica	0/2/3	leaves/salads, soups, spreads	/	[12,83]
	*Leucanthemum* Mill.	ivanjščica, navadni hlapček, volovsko oko, kresnica	0/0/1	flower buds/salads, soups, in vinegar	/	/
	*Matricaria* sp. L. (especially *M. recutita* L.)	maternik, maternjak, kamilica, gamilica, hermanek, kamenika, mrvca, rumenek, gomiljica, vonjavka	8/5/39	inflorescences and leaves/garnishes, beverages, soups, egg dishes	Primorska	[27,42,46,50,69]
	*Scolymus hispanicus* L.	sikalina, bermeč rumeni badelj, brmbeč	0/2/0	young shoots/soups, vegetable and egg dishes	Primorska	[12,85]
	*Scorzonera hispanica* L.	zmijak, črni koren, španski koren	0/2/0	roots/vegetable dishes, salads	Primorska	[12,85]
	*Sonchus* L. (especially *S. oleraceus* L. and *S. asper* (L.) Hill)	škrbinka, mleč	0/4/3	young leaves/soups, salads, vegetable and egg dishes, pastries	Primorska	[12,43,82,85]
	*Tanacetum parthenium* (L.) Sch.Bip.	madrijalica, madrijolica, mandrijana, madrjanca, mandrijerica	4/6/1	leaves/egg dishes, soups	Primorska	[44,57,60,62,67,69,82,85]
	*Taraxacum* F.H. Wigg	jajčar, lederče, mleč, virgrad, solatnik, števnica, mlečje, otavčič, smolika, smolička, farška plata, žehtelnica	71/39/96	leaves and inflorescences/salads, soups, spreads, syrups, beverages, egg, meat and vegetable dishes	Primorska	[21,24,27,30,31,32,36,37,38,39,42,45,50,51,56,60,62,63,64,65,66,67,69,70,71,77,78,79,85]
	*Tragopogon* L.	kozja brada	2/2/5	young shoots, inflorescences/spreads, salads, soups	/	[43,64,65]
	*Tussilago farfara* L.	arpinc, elpejž, lupinec, jurjevka, kopačnica, lepušček, spodbel, stiper, svinjarica, štipor, vinogradska kopačica	1/8/4	Inflorescences, leaves/spirits and salads	/	[2,12,42,43]
Berberidaceae	*Berberis vulgaris* L.	babkovina, česmin, cvič, češniga, češmigovec	3/4/6	fruits/beverages, jams, desserts	/	[42,63]
Betulaceae	*Corylus avellana* L.	leska, leščevje, lešnjik, liškan	96/28/43	seeds/desserts, fruit dishes, pastries	/	[31,36,38,41,42,45,50,51,58,60,63,64,68,78,84,85]
Boraginaceae	*Anchusa officinalis* L.	volovski jezik	0/0/1	leaves/soups	/	/
	*Borago officinalis* L.	boreč, boraga, buraža, poraga, lisičika, lisičina	2/2/3	aerial parts/salads, soups, garnishes	/	[42,43,67]
	*Pulmonaria* L (especially *P. officinalis* L.)	cmulež, pikec, navadna plučnica, kuščernjak, srčnica, zajčeki	1/5/3	leaves/spirits, salads	/	[2,60,63]
	*Symphytum officinale* L.	celivec, sveni, svaljnik, črni koren	1/3/2	young leaves/soups, vegetable dishes	/	[43,50,55,82,85]
Brassicaceae	*Alliaria petiolata* (MB.) Cav. et Grande	česnovka, hostni česen	0/0/2	leaves, young shoots/spreads, salads	/	/
	*Armoracia rusticana* G.Gaertn., B.Mey. & Sch-erb.	hren, turman, ren	103/40/32	roots and leaves/sauces, garnishes, vegetable, meat and egg dishes	/	[21,22,24,27,30,31,32,36,41,44,45,46,51,57,60,63,66,70,80,85]
	*Barbarea vulgaris* W. T. Aiton	barbica	0/3/1	leaves/salads, spreads, garnishes	/	[2,12,85]
	*Camelina sativa* (L.) Crantz.	riček, toter	3/0/0	seeds/bread	/	[60]
	*Cardamine* L.	penuša, konopnica	2/2/12	aerial parts/salads, spreads	/	[12,21]
	*Cardamine amara*	grenka penuša	0/0/1	aerial parts/spreads	/	/
	*Cardamine bulbifera*	brstična konopnica, mlaja	0/0/2	aerial parts/salads, soups, spreads	/	/
	*Cardamine enneaphyllos* (L.) Crantz	deveterolistna konopnica	0/0/1	aerial parts/salads	/	/
	*Capsella bursa-pastoris* (L.) Medik.	divja repica, bobulica, divji srčeki, torbica, plevelka, lažnica, luščec, pucalica, poljska preslica, škofove kapice, škrobotec	2/2/6	leaves/salads, spreads, garnishes	/	[21,60,85]
	*Diplotaxis* DC.	dvoredec, rukulja	3/3/11	leaves/salads, spreads, garnishes	Primorska	[43,62,69,85]
	*Eruca sativa* Mill.	rukvica	6/2/21	leaves/salads, spreads, garnishes	Primorska	[36,50,62,69,82,83]
	*Lepidium* L.	draguša	0/1/0	aerial parts, seeds/as a spice, soups, salads	/	[28]
	*Lunaria rediviva* L.	srebrenka	0/0/1	leaves, flowers/salads, spreads	/	/
	*Nasturtium officinale* W. T. Aiton	navadna kreša, bobovec, drezga, kreša, korešča, potočarka, režnik, studenčnica, vodni dihalnik	2/3/7	shoots and leaves/salads, spreads, sauces, meat dishes	/	[31,43,60,73,85]
	*Sinapis arvensis* L.	gorjušica	0/0/1	aerial parts, seeds/as a spice, soups, salads	/	/
	*Sisymbrium officinale* (L.) Scop.	dihnik, rukulja, lažnica, rumena železnica, svinjek	0/2/1	leaves/soups	/	[43,85]
	*Sisymbrium officinale* (L.) Scop.	dihnik, lažnica, svinek, rukulja, rumena železnica, svinjek	0/3/0	leaves/salads, spreads, garnishes	/	[2,12,43]
Campanulaceae	*Campanula* L.	repušica, zajka, zvončica, ležnjačič	0/3/0	leaves, roots/soups, salads, vegetable dishes	/	[43,85]
Cannabaceae	*Celtis australis* L.	koprivovec, kostilja, koprivca, ladonja, kostela, koščela, fanfarika	0/4/1	fruit/raw, beverages	Primorska	[43,82,84,85]
	*Humulus lupulus* L.	hmelj, falon	4/3/17	young shoots/salads, vegetable and egg dishes	Štajerska, Primorska	[21,52,60,62,63,85]
Capparaceae	*Capparis spinosa* L.	kapra, kapar	0/2/0	flower buds, fruits, leaves/fermented, fish dishes, salads	Primorska	[43,85]
Caprifoliaceae	*Lonicera caerulea* L.	planinsko kosteničevje	0/0/1	fruits/beverages, fruit dishes	Gorenjska	/
	*Valeriana officinalis* L.	baldrijan, špajka	0/0/1	young leaves/salads	/	/
	*Valerianella* Mill.	motovilec	2/5/15	leaves/salads, beverages	/	[31,36,46,63,65,66]
Caryophyllaceae	*Silene vulgaris* (Moench) Garcke	pokalica, mehurjasta lepnica	2/2/5	young shoots and leaves/soups, vegetable, egg and meat dishes	Primorska	[27,32,50,59,69,85]
	*Stellaria* L. (especially *S. media* (L.) Vill, and *S. neglecta* Weihe)	zvezdica, kurja črevca	5/2/17	young shoots/salads, soups, vegetable dishes	Gorenjska, Ljubljana	[55,82,85]
Cornaceae	*Cornus mas* L.	drnulja, dren	4/14/9	fruits/beverages, jams, desserts	Primorska	[22,42,45,60,63,84,85]
Crassulaceae	*Sedum album* L.	bela homulica, homulca	0/0/1	leaves/salads, spreads	/	/
	*Sempervivum tectorum* L.	uhlek, ušesnik, uhlec, gromnik, gromotresk, netresk, strešnik	0/3/0	leaves/salads, vegetable dishes, beverages	Primorska	[43,82,85]
Cupressaceae	*Juniperus communis* L.	čepin, čopin, smrčika, brinjkola, kleka, smrekva, brinje, brina, navadna brina	29/18/16	fleshy cones/spirits, soups, garnishes, meat dishes	Primorska	[2,12,22,29,32,41,42,45,52,55,57,60,64,70,84,85]
	*Juniperus oxycedrus* L.	rjava brinkola, črni brin, brinje	2/4/3	fleshy cones/spirits	Primorska	[65,82,84,85]
Dioscoreaceae	*Tamus communis* L.	blušč, bljušt	3/3/10	young shoots/egg dishes	Primorska	[43,64,65,82,85]
Dryopteridaceae	*Dryopteris filix-mas* (L.) Schott	prava glistovnica, glistna podlesnica	0/0/1	young shoots/soups, egg dishes	/	/
Eleagnaceae	*Hippophae rhamnoides* L.	rakitovec, pasji trn	0/0/1	fruits/beverages, fruit dishes	/	/
Equisetaceae	*Equisetum arvense* L.	konjski rep, hvost, fašec, vošč, štukovac, žabna, rabozel	2/6/2	aerial parts/beverages	Gorenjska, Primorska	[12,42,54,60]
Ericaceae	*Arbutus unedo* L.	jagodičnica, planika, magunja	1/4/0	fruits/spirits	Primorska	[43,69,84,85]
	*Rhododendron hirsutum* L.	gričevnik, kosmati sleč, hudičela, ravš, ravšje	0/0/1	flowers/teas	/	/
	*Vaccinium myrtillus* L.	borovje, črnica, črna jagoda, risnica	35/43/79	fruits/beverages, jams, desserts	/	[2,21,25,29,31,33,40,41,42,45,47,50,52,53,54,56,58,60,63,66,69,70,71,74,78,80,84]
	*Vaccinium vitis-idaea* L.	belke, gorenk, gramzelše, netečje, omanjščevina, mešičevje, močnica, rdeča malenca, rdeče črnice	24/7/16	fruits/beverages, jams, desserts, egg and meat dishes, salads, garnishes, sauces	Štajerska, Koroška	[21,23,29,31,41,42,45,51,52,54,60,66,78,84]
Fabaceae	*Anthyllis vulneraria* L.	boljunec, medvejka, mačkina detelja, mačje tace, uročnik, zajčja detelja	1/0/8	aerial parts/beverages	Gorenjska	[42]
	*Lathyrus* L.	grahor, grašica, divji grah, lot	0/1/0	seeds, tubers/cooked, roasted	/	[12]
	*Robinia pseudoacacia* L.	robinija, akacija	6/15/19	flowers/beverages, desserts, fruit and egg dishes	Primorska	[36,37,45,50,62,66,67,69,75,84,85]
	*Trifolium* L.	detelja	1/1/1	inflorescences/spirits	/	[42,82]
	*Trifolium pratense* L.	travniška detelja	0/0/1	inflorescences/spirits	/	/
	*Trifolium montanum* L.	gorska detelja	0/0/1	inflorescences/nonalcoholic beverages	/	/
Fagaceae	*Castanea sativa* Mill.	kostanj, domači kostanj, kostanje, kostanjevec, kostanjek, kostanja	79/94/87	fruits/spreads, desserts, soups, fruit and meat dishes	/	[2,27,31,32,36,38,41,42,45,50,51,55,59,60,61,63,64,65,78,84,85]
	*Fagus sylvatica* L.	bukva, hiba	2/7/19	young leaves and seeds/salads, beverages, oil	Gorenjska, Primorska, Notranjska	[55,62]
	*Quercus* L. (especially *Q. robur* L., *Q. pubescens* Willd. and *Q. petraea* (Matt.) Liebl.)	hrast, pitnek, pisanec, drobljak, grelc, gnilec, gnjel, gnjilec, ličnik, nitnik, nitnjak	0/10/1	fruits/beverages, pastries, desserts	/	[2,12,43,82,83,84,85]
Gentianaceae	*Centaurium* Hill (especially *C. erythraea* Rafn)	tavžentroža, cintaaver, čintara, glistnik, gorčica, griževec, grenka trava, centaver	3/4/12	aerial parts/beverages, desserts	Štajerska	[42,60,85]
	*Gentiana lutea* L.	cijan, encijan, gencijan, košutnik, gorčica, svedr. Lecjan, srčenjak, svišč, goreuca	2/1/4	roots/spirits	Gorenjska, Primorska, Štajerska	[60,85]
Grossulariaceae	*Ribes* L.	kosmulja, grozdičje, ribez, ribezelj	1/3/0	fruits/beverages, desserts, fruit dishes	/	[39,55,68,69]
Hypericaceae	*Hypericum perforatum* L.	šentjanževa roža, ivanovka, krčnica, krčna zel, janževka, jezusova kri, grilacec	2/0/3	aerial parts/spirits	Ljubljana	[42]
Iridaceae	*Crocus vernus* (L.) Hill	podlesk, uscanka, žafran, kroketi	0/1/1	corms/raw	Primorska	[83]
	*Iris* Tourn. ex L.	perunika	0/2/0	rhizoma/boiled, roasted	/	[43,82]
Juglandaceae	*Juglans regia* L.	oreh, arh, laški oreh	236/127/27	seeds and young leaves/spreads, desserts, soups, fruit, vegetable and meat dishes, pastries, beverages	Primorska	[21,22,23,24,27,28,30,31,32,35,36,38,41,42,44,45,50,51,55,59,60,61,63,64,65,66,68,69,78,80,84]
Lamiaceae	*Betonia officinalis* L.	petonka, bitunica, gušar, rani list, ranjak	0/2/0	leaves/beverages, spreads	/	[12,85]
	*Calamintha* L.	čober, čuber, šetrajnik	0/5/0	aerial parts/beverages, vegetable dishes	Primorska	[2,12,43,82,85]
	*Clinopodium vulgare* L.	čepič, čepac	0/2/0	leaves/spreads, salads	/	[12,85]
	*Glechoma hederacea*	popenec, povojček, srednjak, stračič, divji perpin, vrednjak, zlata ketnica, prisadna zel	0/4/2	leaves and flowers/desserts, soups, salads	/	[2,12,43,85]
	*Hyssopus officinalis* L.	ožep, ožepek	0/3/2	aerial parts/beverages, meat dishes	Primorska	[12,83,85]
	*Lamium* L.	mrtva kopriva, lisavka, pezičevje, žibrt, pivka, prisadence, prisadnik, maronica, žibrat	0/2/7	flowers/salads	Gorenjska	[12,55]
	*Melissa officinalis* L.	aselnica, črniva, čebeloperka, maternjak, medenka, medeni list, melisa, srčno zelje, rojevnica, osenika	19/6/36	leaves/desserts, beverages, egg and vegetable dishes	Primorska	[21,27,35,36,38,39,42,44,45,52,57,60,62,63,67,69,70,71]
	*Mentha* L.	meta, minca, metvica	51/16/47	leaves and inflorescences/desserts, beverages, egg, meat, fruit and vegetable dishes	Primorska	[22,27,29,39,41,42,45,46,50,51,52,58,59,61,63,64,69,85]
	*Mentha pulegium* L.	polaj	2/0/0	leaves/meat dishes	Primorska	[59]
	*Origanum vulgare* L.	dobra misel, bolmet, čober, divji majaron, tošta, zavrta	9/4/19	leaves and inflorescences/beverages, soups, salads, garnishes, egg, meat and vegetable dishes, teas	/	[42,43,52,58,59,60,61,70,85]
	*Prunella* L.	črnoglavka	0/0/1	young shoots, leaves/soups, salads	/	/
	*Salvia officinalis* L.	čistec, kadulja, prava kadulja, žajbelj, žlahtni žajbelj	15/9/50	leaves/beverages, spirits, sauces, garnishes, egg, milk and meat dishes	Primorska	[22,30,32,41,44,45,51,52,59,60,61,63,64,66,67,69,71,84,85]
	*Salvia pratensis* L.	travniška kadulja, čistec, kadulja	2/1/2	leaves/egg dishes, soups	Koroška, Primorska	[22,27,85]
	*Salvia rosmarinus* Spenn.	rožmarin, pravi rožmarin	22/8/57	aerial parts/soups, vegetable and meat dishes, teas, vinegar, spiced oil	Primorska	[21,27,29,32,36,41,42,44,45,50,51,52,57,60,69,70,71,77,84,85]
	*Satureja* L. (especially *S. montana* L.)	šetraj, čober, šatraj, šetrajka	9/5/11	aerial parts/soups, meat and vegetable dishes	Primorska, Notranjska	[22,42,43,52,59,64,70,82,85]
	*Teucrium chamaedrys* L.	vrednik, učnica, komandl, mrzlični koren, krčnica	0/0/1	leaves/egg dishes	Gorenjska	/
	*Teucrium montanum* L.	trava iva, gorska urhovka	0/1/0	leaves/soups, meat and fish dishes	Primorska	[85]
	*Thymus* L.	poljska materina dušica, babja dušica, divji timijan, dušje, mačešica, materinka, preprišč, prežilka, bukvica	17/9/42	aerial parts/beverages, soups, garnishes, teas, egg, meat, fruit and vegetable dishes, desserts	/	[22,32,41,42,45,50,52,58,59,60,61,63,66,67,69,70,82,85]
Lauraceae	*Laurus nobilis* L.	lovorika, lovor, plemenita lovorika	16/9/46	leaves/soups, sauces, meat and fish dishes	/	[22,31,32,38,41,42,44,45,50,52,58,59,60,64,66,70,78,82,85]
Lythraceae	*Trapa natans* L.	vodni oreh, kostanjevec	0/1/0	seeds/roasted	/	[43]
Malvaceae	*Althaea officinalis* L.	ajbiš, beli slez, beli popelj	2/2/3	aerial parts/beverages	/	[12,42,85]
	*Malva* L.	črni klobuk, divji papel, divji slez, slezena, sleznica, divji *slezenovec*, divja, slezena, divja škura, škuvra, škurja, škurva	2/3/5	aerial parts/beverages	Primorska	[69,77,85]
	*Tilia* L.	lipovec, lipa	9/7/38	flowers/beverages, desserts	Primorska	[22,25,27,29,42,46,53,54,55,56,60,66,67,69,71,72,77,82]
Moraceae	*Ficus carica* L.	figa, smokva, smirnska jagoda	20/9/54	fruits/desserts, fruit dishes, pastries	Primorska	[29,44,46,62,64,67,68,69,84]
Myrtaceae	*Myrtus communis* L.	mirta, murta, marta, martina, mrčela	0/2/0	fruits/raw, spirits	Primorska	[84,85]
Oleaceae	*Fraxinus excelsior* L.	belijesen	2/0/0	young leaves/soups		[22]
	*Fraxinus ornus* L.	črni jesen, jasenica	0/1/0	sap/desserts, beverages	Primorska	[85]
	*Olea europaea* L.	divja oljka, maslina, ulika, oliva	0/2/0	fruits/raw, fermented	Primorska	[84,85]
Oxalidaceae	*Oxalis* L. (mainly *O. acetosella* L.)	zajčja deteljica, božji kruhek, cicelj, kisla detelja, rezka detelja, zajčja sol	0/2/4	leaves and flowers/salads, garnishes, fruit dishes	/	[2,12]
Papaveraceae	*Chelidonium majus* L.	aselca, bradavičnik, ceduljka, drafna trava, cengulja, krvnik, dražnica, krivi zelje, rdeči mleček, skrobla, zlata korenina, kačji mleček	0/0/2	leaves/spirits	/	/
	*Papaver rhoeas* L.	prpelica, gospodičnica, križec, divji mak, pumpala, roštalca, putpelica	0/0/1	petals/as a dye for syrups	/	/
Pinaceae	*Abies alba* Mill.	hoja, hojka	0/1/3	young shoot tips/spirits and syrups	/	[12]
	*Larix decidua* Mill.	macesen, viharnik	1/1/3	young shoot tips/spirits and syrups	/	[42,55]
	*Picea abies* (L.) H. Karst.	smreka	8/5/39	young shoot tips/spirits and syrups	/	[27,42,55,66,68,70,71,80]
	*Pinus* L. (especially *P. sylvestris* L., *P. nigra* J. F. Arnold, and *P. mugo* Turra)	bor, borovec, rušje, košutje	3/2/11	young shoot tips and young cones/spirits and syrups	Gorenjska, Primorska	[2,12,42,69]
Plantaginaceae	*Plantago* L.	mačica, mokovec, trpotec, pripotnik, škripotec	5/4/19	leaves and inflorescences/salads, beverages, egg, meat and vegetable dishes	/	[27,29,45,55,77,85]
	*Plantago* lanceolata L.	celec, kozja rebrca, ovčji jezik, ozki protek, krpotec, suličasti trpotec, žebinec, iljak, žilnik	2/3/10	leaves and inflorescences/salads, egg, meat and vegetable dishes	/	[42,60,85]
	*Veronica* L. (especially *V. beccabunga* L. and *V. anagallis-aquatica* L.)	jetičnik, bobovnik, jeterčnik, sorej, veronka, lehtica, bodčec, unje, velnica, lobovnjak, vodni repincelj	0/0/2	leaves, shoots/salads, soups, vegetable dishes	/	/
Poaceae	*Arundo donax* L.	kanela, trstika, kana, trska, rozga	0/3/1	young stalks/beverages	Primorska	[2,82,85]
	*Phragmites australis* (Cav.) Trin. ex Steud.	trst, trstika, bičje, mečiček, ščevar, ševar, vodna trska, trstika	0/1/1	young stems and leaves/desserts, bread	/	[85]
Polygonaceae	*Polygonum* L.	adreselj, drnoselj, glistnjak, kurji jezik, moljava, norava, pernica, ptičji dresen, rakovica, svinjska kaša, trdina, tristovec, troskovec, vrbnica, žabja solata	0/1/2	leaves, shoots/spreads, soups	/	[85]
	*Rumex* L.	kislica, kisavec, smuk	11/6/40	leaves/salads, soups, spreads, sauces, garnishes, egg and meat dishes	Štajerska	[21,27,31,32,41,45,46,51,60,63,72,85]
	*Rumex acetosa* L.	kisavec, ajdovec	0/1/1	leaves/salads, soups, spreads, egg dishes	/	[43]
	*Rumex alpinus* L.	alpska kislica, planinska kislica	0/0/1	young leaves/vegetable dishes	Gorenjska	/
Polypodiaceae	*Polypodium* L.	sladka koreninica, medenična praprot, oslad, sladič, sladka praprot, sladki koren, sladka steljica, šentjanževa korenina	1/2/5	rhizoma/beverages	/	[22,72]
Portulacaceae	*Portulaca oleracea* L.	tolščak, portulak	2/3/3	young shoots/salads, soups, vegetable dishes	Primorska	[43,45,69,85]
Primulaceae	*Primula vulgaris* Huds.	trobentica, ovčica, brkončica, jeglič, golšica, gospodična	5/8/29	young leaves and flowers/salads, beverages, soups, vegetable dishes	/	[21,27,30,50,51,60,69,77,83,85]
Ranunculaceae	*Caltha palustris* L.	jurjevka, šenčurka, kurešnica, paludnica, studenčnik, zlatenica	0/0/1	petals/to dye butter	/	/
	*Clematis vitalba* L.	vezela, bulida, škrobot, trtovičje, leza, trtorina, vezelje	0/2/0	young shoots/egg dishes	Primorska	[43,82]
	*Ficaria verna* Huds.	lopatica, bradavičnik, motika	0/0/1	young leaves/soups, vegetable dishes	/	/
Resedaceae	*Reseda* L.	katanec, reseda, rezeda	0/0/1	leaves/spreads, salads	/	/
Rhamnaceae	*Paliurus spina-christi* Mill.	drača, diraka, derak	0/1/0	fruits/raw, bread	Primorska	[84]
Rosaceae	*Alchemilla* L.	hribska resa, rosnik, plenička, božja plahtica, hlebec	1/5/3	aerial parts/salads, spirits	Gorenjska	[2,12,42,55]
	*Amelanchier ovalis* Med.	skalna hrušica	0/0/1	fruits/beverages, fruit dishes	/	/
	Crataegus Tourn. ex L. (3 species)	beli trn, glag, glagon, glogovec, glaginje, glozje, medvedove hruške	2/6/18	fruits/beverages, jams	Notranjska, Gorenjska, Primorska	[42,55,84,85]
	*Fragaria* L.)	gozdna jagoda, smokvica, trstek, troskva	38/28/91	fruits/beverages, jams, desserts	/	[22,25,28,29,32,33,42,44,46,47,51,54,56,60,66,67,69,79,84,85]
	*Geum urbanum* L.	blažič	0/0/1	roots/spirits	/	/
	*Malus sylvestris* (L.) Mill.	lesnika	2/5/9	fruits/vinegar, beverages, desserts	Gorenjska, Primorska	[2,69,71,78,80,84,85]
	*Prunus avium* L. var. *sylvestris* (Kirschl.) Dierb.	češnja drobnica, črešnja, tičarica	3/6/19	fruits/beverages, fruit dishes, desserts, spirits	Gorenjska, Primorska, Dolenjska	[55,60,71,74,84,85]
	*Prunus cerasifera* Ehrh.	cibora, ringlo, mirobalana, vinjika, divja sliva	0/1/0	fruits/fruit dishes, desserts, beverages	Primorska	[84]
	*Prunus mahaleb* L.	rešelika	0/4/5	fruits/beverages, fruit dishes, desserts	Primorska	[43,82,84]
	*Prunus padus* L.	čemš, čremka, črensa, čremž	0/0/1	fruits/beverages	/	/
	*Prunus spinosa* L.	črni trn, grmulja, divja slivica, oparnica, trnika, trnovec	7/16/20	fruits/beverages, fruit dishes, desserts, spirits, vinegar	/	[26,42,44,55,63,68,69,84,85]
	*Pyrus pyraster* (L.) Burgsd.	divja hruška, drobnica	0/4/3	fruits/beverages, desserts, fruit dishes	Gorenjska, Primorska	[26,55,72,85]
	*Pyrus amygdaliformis* Vill.	mandljevolistna hruška	0/4/0	fruits/raw, beverages	Primorska	[43,82,84,85]
	*Rosa* L.	šipek, babji zob, bavec, divja roža, goščavka, pasja gartroža, srboritka	16/13/52	fruits/beverages, jams, desserts, soups	Primorska	[27,32,41,42,44,46,60,63,67,68,69,74,80,84,85]
	*Rubus* L.	robida, črnina, kopina, črna malina, kopinjak, kopinjek	19/26/87	fruits/beverages, jams, desserts, spirits	/	[25,31,32,41,44,45,55,69,76,80,84,85]
	*Rubus idaeus* L.	malinjek, malinje, muraga, planinka, rdeča kopina, maljoga, žlahtna malenca	52/26/72	fruits/beverages, jams, desserts, sauces, soups, vinegar	/	[23,27,31,32,38,41,42,44,45,54,55,58,60,63,66,74,78,79,80,85]
	*Sanguisorba minor* Scop.	zelena svitlica	0/0/1	young leaves/soups, spreads	/	/
	*Sorbus aria* (L.) Crantz	mokovica, mokovec, mokalica	2/7/6	fruits/fruit dishes, pastries, desserts, beverages	Gorenjska, Notranjska	[44,55,68,70,82,84,85]
	*Sorbus aucuparia* L.	rebika, rebičje, jerebičje, gorska smrdivka, jerebikovec, nedeljski les, smrdlika	3/7/13	fruits/jams, spirits, wine, teas, garnishes	Gorenjska	[12,42,44,55,82,84,85]
	*Sorbus chamaemespilus* (L.) Crantz.	pritlikava nešplja, nešpljica	0/0/1	fruits/raw, spirits	Gorenjska	/
	*Sorbus domestica* L.	skorš, skurša	4/5/3	fruits/spirits, fruit dishes	Primorska	[44,64,69,77,82,84,85]
	*Sorbus torminalis* (L.) Crantz	brek, breka	1/2/10	fruits/jams, spirits, teas, fruit dishes	Primorska	[44,69,84]
Rubiaceae	*Asperula* L.	perla, bulomajster, dišeča strašnica, medenica, prehlajenka, prvenec, rožna perlica, siriščina, želvenica	0/0/1	flowers/beverages, spirits	/	/
	*Galium aparine* L.	plezajoča lakota	0/0/1	young shoots, fruits/spredas, beverages	/	/
	*Galium odoratum* (L.) Scop.	dišeča lakota, dišeča perla	2/0/3	inflorescences/beverages	/	[60,63]
	*Galium verum* L.	divji lan, dremovka, *lakota*, mlekoseda, mrtva torica, obročkovina	2/0/1	inflorescences/beverages	/	[42]
Rutaceae	*Dictamnus albus* L.	jasenjak, beli jasen, diptam, jesenov koren	0/2/0	flowers, roots/spirits	Primorska	[12,85]
	*Ruta* L. (especially *R. graveolens* L.)	rutica, ruta, vendrica, verant, rudo, rutvača, rutvica	0/1/2	leaves/meat dishes, spirits	/	[85]
Sapindaceae	*Aesculus hippocastanum* L.	konjski kostanj, jeloš	2/0/5	seeds/beverages	Primorska	[42]
Scrophulariaceae	*Verbascum* L.	lučnik, papeževa sveča, svečnik	0/0/1	flowers/spirits	/	/
Smilacaceae	*Smilax aspera* L.	oponec, ostri smilaks	0/3/1	young shoots, roots/egg and vegetable dishes	Primorska	[12,43,85]
Solanaceae	*Physalis alkekengi* L.	rdeča punčica, mošnjičnik, pokalin, scalnik	0/0/1	fruits/desserts, fruit dishes	/	/
Staphyleaceae	*Staphylea pinnata* L.	kloček, divji orešek	0/0/1	seeds/raw	Notranjska	/
Taxaceae	*Taxus baccata* L.	tis, tisa	0/0/1	arillus/desserts	/	/
Urticaceae	*Parietaria* L.	krišina, cerkvina, ščirica, ščinjerica, ščirika	0/2/1	leaves, young shoots/soups, salads, egg dishes	Primorska	[43,85]
	*Urtica* L (especially *U. dioica* L.)	kopriva, koprica, ožarnica, pečenica, žagarica, žgavnica, žgoča kopriva, živa kopriva, ožigavica, pokriva	58/20/51	leaves and young shoots/salads, soups, spreads, sauces, garnishes, egg, meat and vegetable dishes	Primorska	[21,27,30,31,32,34,36,41,50,51,52,60,62,64,65,67,68,69,71,77,78,84,85]
Violaceae	*Viola* L.	vijolica, babji stolček, ljubica, fijolica	3/5/7	leaves and flowers/salads, desserts, beverages, vegetable dishes	Štajerska, Primorska	[42,51,64,69,82,85]
Vitaceae	*Vitis vinifera* L.	divja loza, trs	0/2/0	fruits/raw, dried, spirits	Primorska	[84,85]

* The cumulative data combining the selected recipes from traditional cookbooks (C), the mentions from the ethnobotanical literature (E) and the responses acquired in the online and field survey (S). ** Vernacular names of plants drawn from the ethnobotanical literature were supplemented with those used by informants.

## Data Availability

The data presented in this study are available on request from the corresponding author.
